# Electrocardiographic Characteristics in 438 Neonates with Atrial Septal Defects

**DOI:** 10.1007/s00246-023-03324-5

**Published:** 2023-11-01

**Authors:** Anna Maria Dehn, Maria Munk Pærregaard, Anna Sellmer, Sofie Dannesbo, Elisabeth Blixenkrone-Møller, Anne-Sophie Sillesen, Anna Axelsson Raja, Kasper Karmark Iversen, Henning Bundgaard, Alex Hørby Christensen, Vibeke Hjortdal

**Affiliations:** 1 Department of Cardiothoracic Surgery, The Heart Center, Rigshospitalet, Copenhagen University Hospital, Blegdamsvej 9, 2100 Copenhagen, Denmark; 2 Department of Cardiology, Herlev-Gentofte Hospital, Copenhagen University Hospital, Copenhagen, Denmark; 3 Department of Cardiology, The Heart Center, Rigshospitalet, Copenhagen University Hospital, Copenhagen, Denmark

**Keywords:** Atrial septal defect, Electrocardiography, Echocardiography, Neonates, Copenhagen Baby Heart Study

## Abstract

Arrhythmias and electrocardiographic (ECG) abnormalities are common among patients with atrial septal defects (ASDs). We studied a large cohort of neonates with ASDs to investigate whether ECG abnormalities are present at this early stage or develop later, secondary to hemodynamic changes. We analyzed the echocardiograms and ECGs from the Copenhagen Baby Heart Study, a population-based cohort study. We compared ECG characteristics of 438 neonates with secundum ASDs to 1314 matched controls. In subgroup analyses, we investigated whether electrocardiographic characteristics were associated with age at examination. Neonates with ASDs (median age, 11 days; males, 51%) had longer *P*-wave durations (58 vs. 56 ms, *p* < 0.001), PR intervals (100 vs. 96 ms, *p* < 0.001), and a more rightward-shifted QRS axis (116 vs. 114 degrees, *p* = 0.032) compared to controls (median age, 10 days; males, 51%). There were no differences between cases and controls in the *P*-wave area, amplitude, or axis. Subgroup analyses showed that the differences in *P*-wave duration and PR interval were present in neonates examined in the first week after birth. The difference in the QRS axis was not found in neonates examined this early but was found in neonates examined at age two to four weeks. In conclusion, ASDs are associated with ECG changes from the neonatal phase. The *P*-wave duration and PR interval are longer in neonates with ASDs when compared to controls as early as the first week after birth, indicating that these changes are not purely secondary, but that neonates with an ASD have altered cardiac electrical activity.

*ClinicalTrials.gov Identifier* NCT02753348 (April 27, 2016)

## Introduction

Secundum-type atrial septal defect (ASD) is one of the most common congenital heart defects. It is considered a simple cardiac defect, but patients with ASDs have increased mortality [1] and long-term morbidity compared to the background population [2, 3]. Complications occur in patients with an ASD even after spontaneous, percutaneous, or surgical closure [2, 4] and regardless of the size of the ASD [5]. The diagnosis of ASD is often made in late childhood or even adulthood. At the time of diagnosis, there are often several concomitant clinical symptoms and findings such as dyspnea on exertion, palpitations, and recurrent pulmonary infections [6, 7].

Arrhythmia is one of the most frequent complications in patients with ASDs and contributes significantly to increased morbidity and mortality. The arrhythmias are mainly of supraventricular origin, particularly atrial fibrillation or flutter is common [4, 8, 9]. The incidence of arrhythmias in patients with ASDs increases with increasing age and it is reported that the incidence of atrial fibrillation or flutter is 30% in patients with an ASD who are aged 40–60 years [10] and 52% in patients aged ≥ 60 years [11]. Furthermore, patients with ASDs often have abnormalities in the electrocardiogram (ECG). Typical ECG findings in patients with ASDs include alterations in *P*-wave morphology, prolonged PR interval, right-axis deviation, and incomplete right bundle branch block (iRBBB) [12, 13]. ECG abnormalities are found in children with ASD as well [14, 15]. Altered *P*-wave morphology is thought to be related to morphological changes of the atria, especially right atrial enlargement, while iRBBB and right-axis deviation are thought to be a consequence of the right ventricular pressure and volume overload. Thus, the ECG abnormalities are considered secondary to the ASD with a left-to-right shunt that over time will cause a pressure- and volume-overload on the right side of the heart. However, the fact that arrhythmias are seen even in patients with small ASDs and even after closure of the defect raises questions about the etiology and pathophysiology.

We aimed to investigate whether the cardiac electrical system in patients with ASDs is different as early as in the neonatal period. We investigated a large population-based cohort of neonates who had all undergone transthoracic echocardiography and were systematically assessed for ASDs. We described the electrocardiographic characteristics in neonates with ASDs and compared them with matched controls from the same birth cohort.

## Material and Methods

### Copenhagen Baby Heart Study

The Copenhagen Baby Heart Study (CBHS) is a multicenter, prospective, population-based cohort study of neonates with prenatal inclusion. The inclusion period was from April 1st, 2016, to October 31st, 2018, and the study was based at the three largest maternity wards in The Capital Region of Denmark (Copenhagen University Hospitals; Rigshospitalet, Hvidovre Hospital, and Herlev Hospital). The hospitals are public hospitals with free access to medical services for all residents, including pregnancy care, and serve a population with diverse socioeconomic and ethnic backgrounds. All expectant parents were offered inclusion in the study. Details on the CBHS study design have previously been published [16, 17]. Written informed consent was obtained from parents before inclusion.

Included neonates in the CBHS had transthoracic echocardiography (TTE) and ECG performed. Maternal, pregnancy, delivery, and infant characteristics were collected and stored in the CBHS database.

### Study Population

Details about the cohort profile for the Copenhagen Baby Heart Study and data obtained in the study are described in a previous publication [17]. We included neonates from CBHS where the TTE included visualization of the atrial septum in 2D with and without color Doppler and with adequate image quality to determine the presence or absence of an ASD [18]. We excluded neonates with concomitant major cardiac malformations, neonates with syndromes, and neonates where no ECG was obtained or where the cardiac examination (TTE and ECG) was performed more than 30 days after birth.

Neonates with ASDs were matched 1:3 to controls from the same birth cohort in CBHS. Matching was performed based on sex, gestational age at birth, and age, weight, and length at the time of examination.

### Echocardiography

#### Acquisition

Transthoracic echocardiograms for neonates included in the CBHS were performed by physicians or sonographers trained in pediatric echocardiography. We used Vivid E9 ultrasound equipment (GE Healthcare, Horten, Norway) with 12 MHz and 6 MHz cardiac sector transducers. Images and measurements were acquired in accordance with the American Society of Echocardiography’s Guidelines for Pediatric echocardiography [19]. The CBHS echocardiographic protocol included standard sub-xiphoid, apical, left parasternal, and suprasternal views [16].

#### Analysis

Echocardiograms were stored and analyzed using EchoPAC software (GE Healthcare, Horten, Norway). Echocardiograms were individually and systematically assessed for secundum-type ASDs by physicians trained in pediatric echocardiography using a novel algorithm for the assessment of interatrial communications in the oval fossa that was developed and validated in the CBHS and has been described in detail previously [18]. Analyses were based on several echocardiographic criteria, where we defined neonates as having an ASD in cases where color Doppler flow was crossing the atrial septum and 2D cross-sectional images showed either multiple visible communications (fenestrated ASD) or one single visible communication with either a diameter of ≥ 4 mm or a location in the inferior part of the atrial septum.

### Electrocardiography

#### Acquisition

Electrocardiograms were recorded after the echocardiograms, while the newborn was calm or sleeping. ECGs were recorded using a MAC 5500 HD system (GE ECG System, Milwaukee, USA) with a paper speed of 25 mm/sec, sensitivity at 10 mm/mV, a sample rate of 500 samples per second, and a bandwidth filter of 0.16–150 Hz. Recordings included lead I, II, III, aVR, aVL, aVF, V1, and in most cases V6.

#### Analysis

All tracings were acquired digitally, and ECG intervals, amplitudes, areas, and durations were automatically analyzed using GE Healthcare Marquette 12SL ECG Analysis Program [20]. ECGs were stored in the MUSE ECG management software (Version 8, GE Healthcare, Milwaukee, USA). The ECG data were validated by medical doctors to ensure adequate data quality, as previously described [21, 22].

We analyzed the following standard ECG parameters: heart rate (beats per minute), PR interval (ms), QRS duration (ms), QRS axis (degrees), and maximum *R*- and *S*-wave amplitudes in lead V1 and V6 (μV). For a specific assessment of *P*-wave characteristics, we investigated the *P*-waves in lead V1 in detail. Lead V1 was chosen as it typically has a prominent *P*-wave while at the same time being relatively robust to movement artifacts. *P*-wave variables included the *P*-wave axis, *P*-wave area, and *P*-wave/*P*′-peak amplitudes. These *P*-waves measurements were calculated utilizing an interpolated line between *P* onset and *P* offset. A second deflection of the *P*-wave was annotated as *P*′, *P*-wave area was the net area of *P* and *P*′, where negative values were subtracted from positive values. *P*-wave duration was calculated as the sum of the duration of the *P* and *P*′-wave, if applicable.

### Statistical Analyses

Categorical data are presented as absolute numbers (percentages) and continuous data are presented as median values and interquartile ranges (IQR). Comparisons between groups (neonates with ASDs and matched controls) were performed using the Wilcoxon rank sum test for continuous ECG variables and by chi-square test for categorical variables. Subgroup analyses were performed according to age at examination; 0–7 days old (week 0–1) and 8–30 days old (week 2–4). *R* statistical software v. 1.2.1335 (Boston, MA, USA) was used for statistical analyses and for matching cases with controls. A *p*-value < 0.05 was considered statistically significant.

## Results

### Study Population

After applying the inclusion- and exclusion criteria, we identified 438 neonates with a secundum ASD according to the used algorithm for the assessment of interatrial communications. The median age at examination was 11 days (IQR 7–15), 223 (51%) were boys and the median gestational age at birth was 40.1 weeks (IQR 39–41). We matched the identified cases 1:3 with controls from the CBHS cohort without ASD. Descriptive characteristics for neonates with ASD and matched controls are shown in Table 1. Measurement of the diameter of the ASD according to the used diagnostic algorithm was feasible in 205 of the included neonates. The mean diameter of the ASDs was 4.5 mm (range 4–10 mm).

**Table 1:** Descriptive characteristics for neonates with atrial septal defects and matched controls

	Neonates with ASD (*n* = 438)	Controls (*n* = 1314)
Sex, male, *n* (%)	223 (51%)	671 (51%)
Gestational age at birth, days	281 (273–287)	281 (273–287)
Age at examination, days	11 (7–15)	10 (7–14)
Weight at examination, kg	3.6 (3.3–4.0)	3.6 (3.3–4.0)
Length at examination, cm	52.5 (51–54)	52.5 (51–54)

Categorical variables are displayed as absolute numbers (percentages) and continuous variables as median values (interquartile ranges)

### Overall Electrocardiographic Findings

The median heart rate was 143 bpm (range 82–214) for neonates with ASDs and 140 bpm (range 71–207) for controls, *p* = 0.11.

We found differences between cases and controls in *P*-wave duration in lead V1, the PR interval, and the QRS axis: Neonates with ASDs had longer *P*-wave durations (58 vs. 56 ms, *p* < 0.001); longer PR intervals (100 vs. 96 ms, *p* < 0.001), and a more rightward-shifted QRS axis (116 vs. 114 degrees, *p* = 0.032) when compared to controls (Table 2). There were no differences between neonates with ASDs and controls with regard to the other *P*-wave-related variables in lead V1, i.e., the area of the *P*-wave, the peak amplitude of the *P*-wave, or the *P*-wave axis. Likewise, there was no difference between cases and controls when investigating QRS durations, or maximum *R*- and *S*-wave amplitudes in lead V1/V6 (all *p* > 0.05; Table 2).

**Table 2:** ECG characteristics for neonates with atrial septal defects compared to controls

	Neonates with ASD (*n* = 438)	Controls (*n* = 1314)	*p*-value
Heart rate, bpm	143 (128–160)	140 (127–156)	0.11
*P*-wave axis, degrees	62 (55–70)	62 (54–68)	0.13
*P*-wave area V1, μV*ms	70 (16–137)	69 (21–127)	0.47
*P*-wave peak amplitude V1, µV	73 (39–112)	73 (43–102)	0.46
Biphasic *P*-wave V1, *n* (%)	146 (33%)	425 (32%)	0.72
*P*′ peak amplitude V1, µV	− 43 (− 53 to − 30)	− 39 (− 53 to − 29)	0.81
*P*-wave duration V1, ms	58 (54–62)	56 (52–60)	** < 0.001**
PR interval, ms	100 (92–106)	96 (90–104)	** < 0.001**
QRS axis, degrees	116 (103–138)	114 (102–133)	**0.032**
QRS duration, ms	56 (54–60)	56 (52–60)	0.06
Max amplitude R-V1, µV	1215 (840–1660)	1157 (805–1584)	0.05
Max amplitude S-V1, µV	693 (322–1074)	629 (351–1010)	0.41
Max amplitude R-V6, µV	888 (625–1210)	903 (625–1245)	0.78
Max amplitude S-V6, µV	639 (452–981)	649 (424–991)	0.10

Categorical variables are displayed as absolute numbers (percentages) and continuous variables as medians (interquartile ranges). *P*-values < 0.05 are considered statistically significant and are marked in bold

### Electrocardiographic Findings in Subgroups Stratified According to Neonatal Age

To investigate whether differences in ECG characteristics between neonates with ASDs and controls were present at birth or developed during the first month of life, we divided the cohort into two subgroups based on the neonates’ age at examination. Specifically, we compared neonates with ASDs examined during the first week after birth (0–7 days old) with neonates from the control group examined at the same age. Likewise, we compared neonates with ASDs examined at age two to four weeks (8 to 30 days old) with controls (Table 3).

**Table 3:** ECG characteristics for subgroups on age at examination: 0–1 week and 2–4 weeks

	Age at examination 0-1 week (0–7 days) *n* = 547			Age at examination 2–4 weeks (8–30 days) *n* = 1205		
	ASD (*n* = 136)	Controls (*n* = 411)	*p*-value	ASD (*n* = 302)	Controls (*n* = 903)	*p*-value
Heart rate, bpm	130 (117–141)	130 (117–143)	0.86	148 (134–164)	146 (132–160)	**0.03**
*P*-wave axis, degrees	63 (54–71)	63 (54–69)	0.46	62 (55–70)	61 (54–68)	0.19
*P*-wave area V1, μV*ms	83 (13–158)	79 (29–146)	0.73	63 (18–132)	63 (15–119)	0.24
*P*-wave peak amplitude V1, µV	83 (41–120)	78 (50–117)	0.91	63 (39–107)	68 (39–97)	0.30
Biphasic *P*-wave V1, *n* (%)	46 (33%)	143 (35%)	0.07	100 (33%)	282 (31%)	0.07
*P*′ peak amplitude V1, µV	− 48 (53 to − 35)	− 39 (− 53 to − 29)	0.061	− 39 (− 49 to − 29)	− 43 (− 53 to − 30)	0.305
*P*-wave duration (*P* + *P*′) V1, ms	60 (56–64)	56 (52–62)	** < 0.001**	56 (52–62)	56 (52–58)	** < 0.001**
PR interval, ms	100 (94–106)	96 (90–104)	**0.014**	100 (92–106)	96 (90–104)	** < 0.001**
QRS axis, degrees	122 (107–150)	121 (107–141)	0.97	115 (103–134)	111 (100–128)	**0.0079**
QRS duration, ms	56 (52–58)	56 (52–59)	0.37	58 (54–60)	56 (52–60)	**0.0055**
Max amplitude R-V1, µV	1459 (927–1918)	1344 (933–1776)	0.179	1137 (810–1538)	1054 (751–1445)	0.09
Max amplitude S-V1, µV	830 (395–1293)	805 (410–1222)	0.813	634 (273–1025)	571 (322–922)	0.30
Max amplitude R-V6, µV	810 (552–1174)	898 (615–1245)	0.242	906 (672–1210)	906 (628–1241)	0.69
Max amplitude S-V6, µV	649 (517–1118)	791 (466–1178)	0.468	639 (419–932)	610 (415–906)	**0.010**

Categorical variables are displayed as absolute numbers (percentages) and continuous variables as medians (interquartile ranges). *P*-values < 0.05 are considered statistically significant and are marked in bold

In the subgroup of neonates examined during the first week after birth (median age at examination 4 days, IQR 2–6), there were no differences in the QRS axis or QRS duration between neonates with ASDs and controls. The longer *P*-wave duration and PR interval in neonates with ASDs compared to controls, however, was found in this subgroup of neonates examined in the first week after birth (60 vs. 56 ms, *p* < 0.001 and 100 vs. 96 ms, *p* = 0.014).

**Fig. 1:**
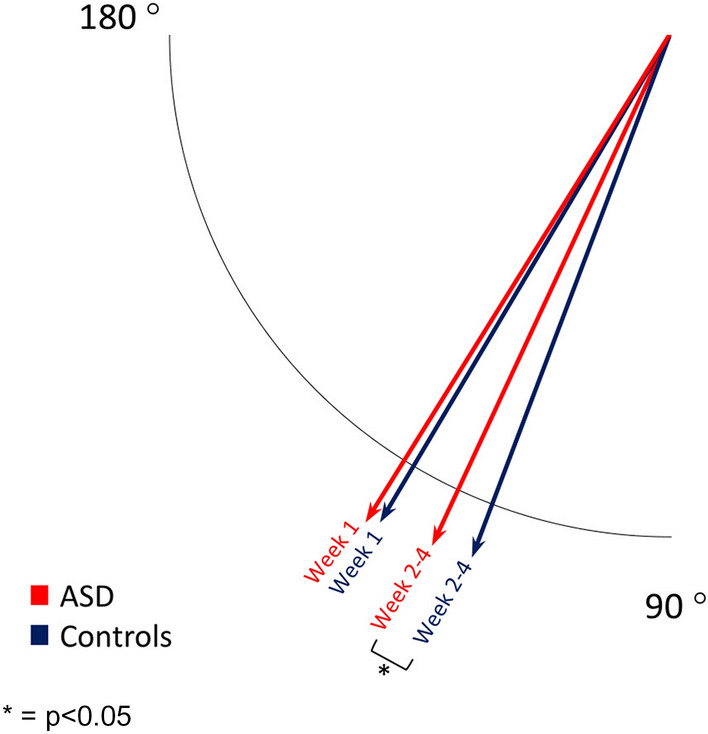
The QRS axis for neonates with atrial septal defects and controls, divided into subgroups by age at examination. The figure illustrates the gradual leftward shift of the QRS axis from week 1 to week 2–4 after birth. Arrows depict median values for the QRS axis in neonates with an ASD (red arrows) and controls (blue arrows). In week 1 after birth, both neonates with ASD and controls have a QRS axis that is physiologically shifted rightwards. During weeks 2–4 after birth, the QRS axis of neonates with ASD stays significantly more rightwards shifted than the QRS axis of controls. * = *p* < 0.05

In neonates examined at age two to four weeks (median age at examination 13 days, IQR 10–17), the *P*-wave duration and PR interval were also longer in neonates with ASDs compared to controls. The difference in the QRS axis with a more rightward-shifted axis in neonates with ASDs than controls was likewise found in the subgroup of neonates examined at age two to four weeks and the difference in this group was more pronounced with a median value for the QRS axis of neonates with ASDs of 115° (IQR 103–134) vs. 111° (IQR 100–128) for controls, *p* = 0.0079 (Fig. 1). In this subgroup, we also found the QRS duration to be longer in neonates with ASDs compared to controls (58 vs. 56 ms, *p* = 0.0055).

Neonates with ASDs examined at age two to four weeks had a higher heart rate than controls (148 vs. 146 bpm, *p* = 0.03) and a higher maximum amplitude of the *S*-wave in V6 (639 µV vs. 610 µV, *p* = 0.010).

Equally to the analyses for the whole cohort, other ECG variables did not differ between the ASD and the control subgroups for either age at examination.

## Discussion

We investigated a large population-based cohort of neonates who have all undergone echocardiography and electrocardiography during the first month after birth and where echocardiograms have been systematically assessed for ASDs using a validated algorithm. Our results show that neonates with ASDs had a longer *P*-wave duration and a longer PR interval than controls. These differences were already seen during the first week of life. Also, neonates with ASD had a QRS axis that was more rightward shifted than in controls, but this was not present until the second to fourth week after birth.

*P*-wave variables in patients with atrial septal defects differ from healthy controls, with longer *P*-wave duration being a common finding [23–25]. However, most studies include only adult patients or patients who have undergone either surgical or percutaneous closure of the ASD. In 47 pediatric patients with ASDs (mean age, 5.3 years; range, 1 month–17 years), right ventricular conduction delay was seen in 78% and right-axis deviation in 58% of the children. The *P*-wave reflects atrial conduction; and prolongation of the *P*-wave has been shown to be associated with arrhythmias, especially atrial fibrillation [26–28]. The pathophysiology behind the alterations in atrial conduction seen in ASD patients has been subject to debate. Altered atrial conduction due to atrial dilatation caused by left-to-right shunting has been the predominant hypothesis but emerging evidence points toward abnormal electrical conduction as an independent primary disease mechanism in ASD patients. Thilén et al. found prolonged *P*-wave duration in adult patients with ASD compared to controls without differences in right or left atrial size, suggesting a conduction delay in ASD patients irrespective of atrial morphological changes [29]. O’Neill et al. found a greater burden of atrial fibrosis in patients with a secundum ASD and found especially right atrial fibrosis to be associated with the presence of arrhythmias in these patients [30].

Our results show that the *P*-wave duration and the PR interval are longer than in matched controls as early as the first week of life. His bundle electrograms have documented prolonged PR intervals in children with ASD [31], which is also in accordance with our findings.

The QRS axis on the ECG is reflecting the average direction of the ventricular depolarization and right-axis deviation is one of the typical findings on the ECG in patients with ASD [14]. In neonates, however, a rightward-shifted QRS axis is normal [32]. During the fetal state, the pulmonary vascular resistance is high, and the right ventricle is the dominant ventricle. After birth, the systemic vascular resistance rises while the pulmonary vascular resistance falls, making the left ventricle gradually more dominant. A substudy from the CBHS showed the gradual leftward shift of the QRS axis in neonates during this transition [33]. Interestingly, we found that the QRS axis in neonates with ASD changed less toward the left compared to controls during the first four weeks after birth. This could indicate that the shunt across the atrial septum in neonates with ASD has implications on either the electrical activity or the structure of the ventricles at this early stage.

In the subgroup of neonates examined at age two to four weeks, we found a higher maximum amplitude of the *S*-wave in V6 in the ASD group compared to controls. Also, the maximum amplitude of the *R* wave in V1 in the ASD group was higher than in controls, though not statistically significant. Both these ECG findings are known to be associated with right ventricular hypertrophy [34]. We also found a longer QRS duration in the ASD group at age two to four weeks. Thus, taken together, our findings indicate that the ASD has implications on the right ventricle after only a few weeks of postnatal circulation. In the subgroup examined at age two to four weeks, the heart rate is higher in the ASD group compared to controls. This could physiologically support the assumption that the ASD has implications on cardiac function this early: increased chronotropy maintains cardiac output despite a left-to-right shunt across the ASD.

We have previously presented the echocardiographic characteristics of neonates with ASDs within the CBHS study cohort [35]. We found that neonates with ASDs (*n* = 716) had larger right ventricular (RV) dimensions (RV longitudinal dimension end-diastole: 27.7 mm vs. 26.7 mm; RV basal dimension end-diastole: 14.9 mm vs. 13.8 mm; and RV outflow tract diameter 13.6 mm vs. 12.4 mm, all *p* < 0.001) as well as larger atrial volumes than matched controls (right atrial end-systolic volume: 2.9 ml vs. 2.1 ml; and left atrial end-systolic volume 2.0 ml vs. 1.8 ml, both *p* < 0.001). Left ventricular dimensions and function did not differ between neonates with ASDs and controls. Hence, there seems to be some association between morphology and electrocardiographic alterations in our cohort of neonates, where *P*-wave duration and PR interval might be related to atrial morphology and rightward shift of the QRS axis might be related to larger right ventricular dimensions. However, as prolonged *P*-wave duration has likewise been shown in adult patients with ASD without atrial enlargement [29], there might also be a component of electrocardiographic abnormalities irrespective of morphological alterations.

Arrhythmias contribute to morbidity in patients with ASDs. Udholm et al. [5] investigated 151 adult patients (mean age 32 years) with small ASDs that were left unrepaired. Despite 80% of the defects had spontaneously closed, 7 days of Holter recording revealed a high prevalence of occult arrhythmias. Our results substantiate the assumption that there is a burden of asymptomatic electrocardiographic alterations in patients with ASD and that the morphological defect in the atrial septum might not be the sole mechanism for morbidity in ASD patients. Current guidelines for the management of ASD patients suggest that ECG recording is included in the routine follow-up of patients with ASD [36, 37]. Our findings emphasize this recommendation. Physicians need to be aware of the risk of covert electrocardiographic abnormalities and arrhythmias in patients with ASD.

One limitation of our study is the fact that we only have one single TTE and ECG for the neonates, while serial ECGs and TTEs could have provided additional information. Also, at this point, we do not have information on the follow-up of the neonates with ASD examined in the study. The neonates were included prenatally and at the time of echocardiographic assessment, most neonates did not have any symptoms suspicious of ASD, but the ASDs were diagnosed because of the echocardiographic examination and systematic assessment for ASD in this large population-based cohort. Interatrial communications are frequently seen in small children, and we do not know which ones will develop hemodynamic and clinical significance. Complications and comorbidities are found in patients with ASD after spontaneous, surgical, or percutaneous closure of the defect and even in patients where the defect is considered so small that no intervention or follow-up is needed [38]. To our knowledge, we are the first to describe electrocardiographic changes in asymptomatic neonates with ASDs from a large population-based cohort, which we consider a particularly interesting, and somewhat surprising, finding. This new observation may point toward an early indication of a later problem.

The differences in electrocardiographic parameters between newborns with ASD and controls found in this study are relatively small in absolute numbers. Electrocardiographic values for neonates with ASD in our study cohort are still within age-specific reference values [32, 34]. Hence, we are careful not to describe the PR interval and *P*-wave duration as prolonged, but solely conclude that these parameters are longer in neonates with ASD than in matched controls. Likewise, the QRS axis is not pathologically rightwards shifted in our cohort but is still significantly more rightward shifted in neonates with ASD than in the control group and remains so during the first month. Even if the differences found in this study are relatively small, they do reach convincing statistical significance due to the large sample size. A strength of our study is that we present data on the largest population-based cohort to date systematically assessed for ASD and thus are aware of the diagnosis of ASD this early after birth. The findings cannot be applied directly to a clinical context, but our results contribute with new knowledge on the electroanatomic understanding of ASDs.

## Conclusion

Neonates with an ASD had a longer duration of the *P*-wave and a longer PR interval than controls; the difference was found as early as in the first week after birth. The QRS axis of neonates with an ASD remained more rightward shifted during the first four weeks of life than the QRS axis of controls. Our findings suggest that the electrical cardiac system in patients with ASDs is abnormal even before the shunt leads to significant structural alterations. This raises the question, of whether the ECG changes can be described as secondary to hemodynamic changes in patients with ASD, or if the altered electrical activity is an independent disease pattern in ASD. Arrhythmias contribute significantly to the morbidity in patients with ASD, and even though we miss the link between these early electrocardiographic abnormalities and late arrhythmias, our results emphasize the hypothesis that arrhythmias in patients with ASD have a multifactorial etiology.

## Data Availability

The data that support the findings of this study are not publicly available due to the privacy of research participants. The data will be shared upon reasonable request to the corresponding author.
